# Implementing alternative estimation methods to evaluate the reliability of Likert-scale instruments

**DOI:** 10.4069/whn.2024.03.12

**Published:** 2024-03-29

**Authors:** Chang Gi Park

**Affiliations:** Department of Population Health Nursing Science, University of Illinois Chicago, Chicago, IL, USA

## Introduction

For the unbiased evaluation of the construct validity of Likert-scale instruments by nursing researchers, polychoric correlation is strongly recommended over Pearson correlation or covariance-based confirmatory factor analysis (CFA) [1]. Similarly, when estimating the reliability of these instruments, the use of polychoric correlation should be considered. Cronbach’s alpha has been observed to underestimate the inter-item reliability of Likert-type ordinal item scales. As a result, the ordinal alpha coefficient, based on the polychoric correlation coefficient, has been introduced for a more accurate estimation of reliability [2,3]. Since the introduction of ordinal alpha, it has gained acceptance as a reliability measure for ordinal categorical item scales [4-9]. Alternative reliability measures for ordinal items have also been proposed [10,11]. However, the application of these reliability estimation methods in nursing studies remains limited, as does the available guidance for nursing researchers regarding how to apply these methods.

Consequently, the purpose of this manuscript is to evaluate the current practice of relying solely on the Cronbach's alpha for the assessment of ordinal Likert-scale instruments. Additionally, this article aims to introduce alternative methods for estimating reliability coefficients, address practical issues associated with the use of software for these estimation approaches, and demonstrate the application of these programs. Although methods other than Cronbach’s alpha may be suitable for estimating reliability, nursing researchers may lack sufficient information regarding their implementation.

## Alternative reliability estimation methods for Likert scales

### Polychoric correlation coefficient as a replacement for Pearson correlation or covariance matrix as an alpha input

The standard formula for the reliability alpha coefficient is not appropriate for ordinal item Likert scales, as Pearson correlation and covariance tend to underestimate associations for ordinal variables. The ordinal alpha coefficient, which estimates reliability based on a polychoric correlation matrix, is the most commonly used measure of reliability for Likert scales [2].

Gadermann et al. [3] used a polychoric correlation matrix to demonstrate the use of polychoric correlation coefficients and factor loading coefficients derived from factor analysis. The average factor loading coefficient was utilized to calculate ordinal alpha. This calculation was demonstrated using R software (R Foundation for Statistical Computing, Vienna, Austria). However, the ordinal alpha coefficient has been regarded as a consistent measure of theoretical reliability [3]. Since the estimation of the polychoric correlation coefficient relies on the latent interval scale (rather than the actual raw item scores) of the underlying ordered item scores, the ordinal alpha reliability coefficient may be overestimated [12].

### Structural equation modeling-based reliability coefficient estimation

Because ordinal alpha is defined based on the polychoric correlations of latent items, it is considered to represent theoretical reliability [2,11,13]. To estimate the association of the actual item-score covariance, researchers have introduced a nonlinear structural equation modeling (SEM)-based reliability estimation method [2,3] for ordered categorical items. This method utilizes the estimated item thresholds, factor loading coefficients, and polychoric correlation matrix derived from the CFA model [6,11]. To compute the ordinal alpha, it is necessary to use model-based population covariances for the categorical scale score.

### Other reliability estimation methods

The primary limitation of the ordinal alpha method, which is based on the polychoric correlation coefficient, is that polychoric correlation measures the association between underlying latent variables rather than the actual scale. Consequently, ordinal alpha is an unsuitable measure of reliability for Likert scales with ordered items [11,13]. As alternatives, nonparametric correlations such as Spearman correlation and the Cramer V have been recommended for assessing relationships between ordered categorical variables [13].

Deflation-corrected estimators of reliability (DCER) methods offer another alternative for reliability estimation by applying nonparametric item-score associations to the reliability estimation formula [14-16]. A primary cause of reliability coefficient underestimation is the biased Pearson item-score correlation used by traditional reliability estimators. By employing nonparametric item-score correlations, this bias can be corrected. DCER methods can be used to calculate alpha and omega by incorporating nonparametric association estimation techniques, such as the Goodman adjusted item-score correlation coefficient, Kruskal gamma, and Sommer D.

Item response theory (IRT)-based reliability estimation [6] is another approach for estimating the reliability of ordinal item Likert scales. This approach was demonstrated in a recent study that introduced Kuder–Richardson formula 20 (KR-20) and 21 (KR-21) in the context of Likert scale data [17].

## Application of reliability coefficient for ordinal item scales in nursing research

Since the first application of ordinal alpha in a nursing study [18], several articles have reported the use of ordinal alpha [19-22] and ordinal theta [23]. For the model-based reliability assessment of ordinal item scales, both the estimated ordinal alpha and composite reliability of Likert-scale instruments have been reported, utilizing coefficients from CFA [24]. Additionally, the application of a nonlinear SEM reliability method employing the semTools R library has been described [25-27]. The limited use of ordinal alpha and nonlinear SEM reliability coefficients in the evaluation of Likert scales within nursing research may stem from an absence of detailed guidance regarding why and how nurse researchers should implement these alternative approaches.

## Software

A straightforward method for estimating ordinal alpha is available through an online program accessible at https://r-apps.shinyapps.io/shinyapps/. Users simply upload a data file, with multiple supported formats that include text, .txt, .csv, .sav, .xls, .xlsm, and .xlsx. The output includes the estimated polychoric correlation matrix and the ordinal alpha reliability coefficient, as well as both the raw and standardized alpha and the reliability of each item when dropped.

A second easily implementable method for assessing ordinal reliability involves the Jamovi PPDA library. The R interface provided by Jamovi enables the use of R libraries without the need to directly operate the R program. Jamovi’s PPDA library—which stands for Psychometrics & Post-Data Analysis [28]—can estimate ordinal reliability coefficients such as alpha and omega, as well as yielding the polychoric correlation matrix.

Other programs for estimating ordinal alpha are R-based and thus require a basic understanding of R to operate.

• R_ufs: This program is the updated version of R_userfriendlyscience. Its scaleStructure() function can automatically detect ordinal categorical variables and calculate reliability coefficients under both interval and ordinal scale assumptions. When invoked without data, scaleStructure() triggers a pop-up window that allows the user to select a data file for reliability coefficient estimation. Depending on the version of R being used, the results may not be displayed on the screen; if this occurs, executing a plot.new() command before running scaleStructure() can resolve the issue. When data are loaded in the R session, entering scaleStructure(dataset name) will present the ordinal alpha estimation, which includes the alpha coefficient based on the interval scale assumption along with omega coefficients.

• R_misty: The item.alpha (dataset name, ordered=TRUE) function is specifically used to determine the ordinal alpha coefficient. The function’s output also includes the estimated polychoric correlation matrix.

• R_psych: When the omega function is used with the poly=TRUE option, the ordinal alpha coefficient can be estimated. The output also contains estimated results for the ordinal version of G.6, omega, omega hierarchical (omega H), omega H asymptotic, and omega total. The alpha function can estimate ordinal alpha when given a polychoric correlation matrix as an input. To generate this matrix, the polychoric(dataset) function must first be applied to the dataset. Once the estimated polychoric correlation matrix is obtained, it can then be used as input for the alpha function.

• R_semTools: The reliability function within the semTools library provides estimates of the alpha and omega coefficients [29]. When used along with the CFA function from the lavaan R library cfa (using the option ordered=TRUE), this function yields ordinal alpha and omega coefficients. The compReISEM() function represents an updated version of the reliability function, designed for specific types of estimation. In particular, it allows for the estimation of ordinal reliability, with or without correction for score variance, through the use of the ord.scale and tau.eq options. When ord.scale=TRUE and tau.eq=TRUE, the resulting coefficient is equivalent to the alpha coefficient. However, when ord.scale=FALSE, the reliability coefficient corresponds to the ordinal alpha value. The lrv2ord function can generate a model-estimated population covariance and correlation matrix. This, in turn, facilitates the estimation of the alpha coefficient using the alpha formula.

• DCER approach: This set of calculation methods has been introduced through an Excel worksheet, with detailed step-by-step instructions presented in the corresponding papers [14-16].

• R_irtreliability: IRT-based reliability programs can be utilized for Likert scales [30]. The R_irtreliability program leverages the results from the R mirt package. The options “mrc” and “trc” are used to estimate the marginal and test reliability coefficients, respectively. The mirt package also includes the empirical_rxx function, designed for the calculation of the reliability coefficient. The R source syntax for estimating reliability using the KR-20 and KR-21 methods, tailored for Likert scale reliability [17], is available at https://osf.io/rk5e2/.

## Demonstration of reliability coefficient estimation methods

To illustrate the ordinal alpha approach and the other alternative reliability estimation methods, the first five items from the dataset bfi (the Big Five Inventory 25 personality items) were utilized as a single factor. This represents the most frequently accessed dataset available from the ‘psych’ R library.

In the R session:

>library(psych)

>library(ufs)

>data (bfi)

>data<-bfi[,1:5]

>data<-invertitems(data,1) for reverse coding for the first item.

**Figure 1.**
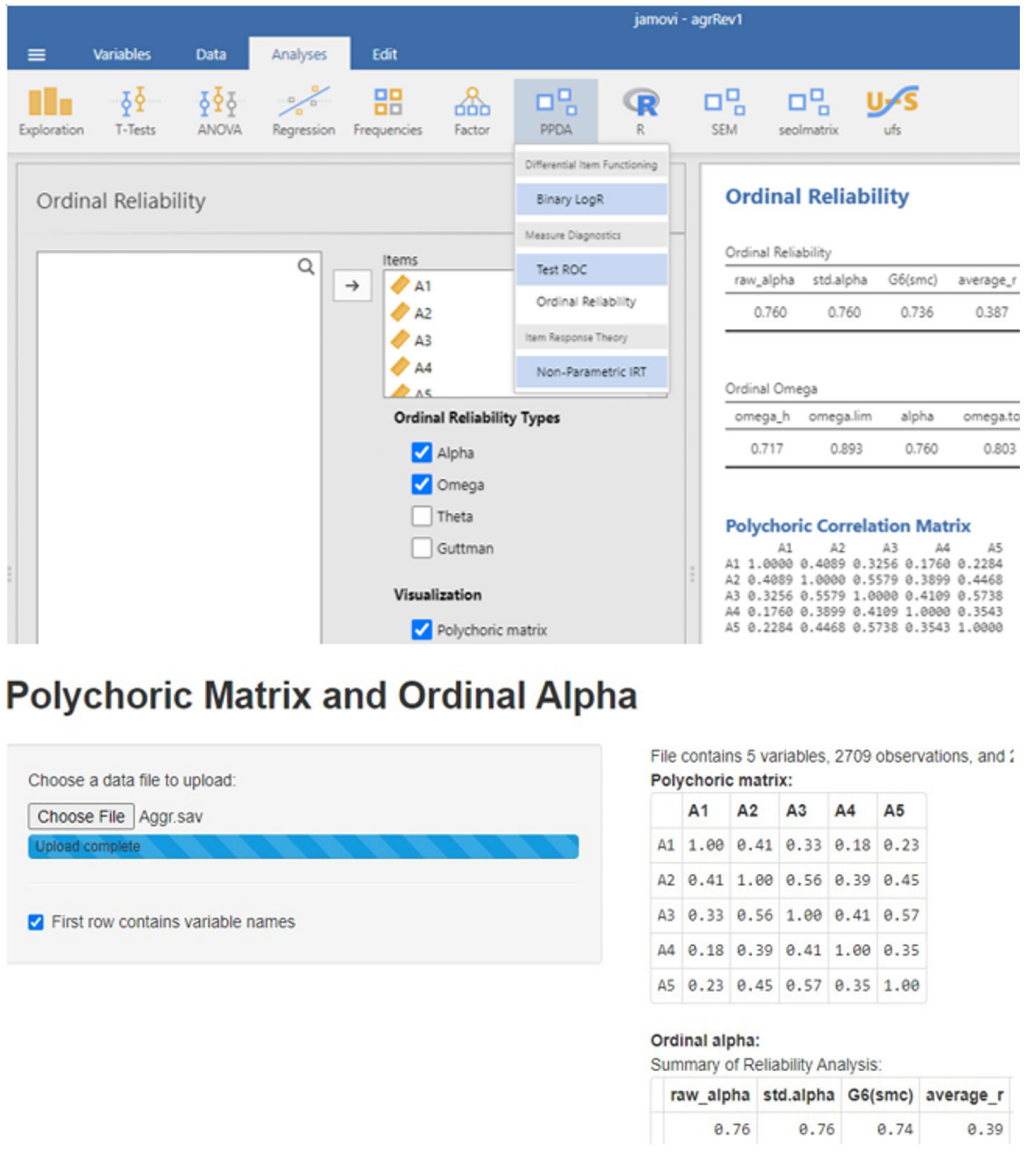
Estimation of ordinal alpha using Jamovi software and an online program.

The application of ordinal alpha estimation can be performed using either an online program or Jamovi software. Two examples of ordinal alpha estimation are presented in Figure 1. The process is straightforward and readily accessible for most nursing researchers.

Table 1 shows the reliability coefficient estimations for the Likert scale, as demonstrated with the sample data. Previous studies have demonstrated methods for ordinal reliability estimation using the R libraries psych, ufs, and semTools [3,7,8].

### How SPSS users can estimate ordinal alpha

The only method available for estimating ordinal alpha within SPSS is to utilize the R program. However, users can also employ the polychoric correlation function HETCOR without directly using R. This function can be added to the SPSS menu by installing SPSSINC_HETCOR.spe, which is available for download at https://github.com/IBMPredictiveAnalytics/SPSSINC_HETCOR. This installation enables the estimation of polychoric correlation coefficients. Once installed, a new menu item, labeled “heterogeneous correlation,” will appear under the “correlation” menu. The polychoric correlation matrix generated by running this function can be copied into an Excel worksheet. In Excel, the average correlation coefficient can be calculated. This average polychoric correlation coefficient is then inserted into the alpha formula: α=k*®r/(1+(k−1)*®r), where *k* represents the number of items and  ®r  denotes the average correlation coefficient. For the sample data, the average bivariate polychoric correlation coefficient was 0.387, resulting in a reliability calculation of 0.7594 based on the alpha coefficient formula. Alternatively, the alpha coefficient can be calculated using other bivariate correlation coefficients, such as the Spearman rho and the Kendall tau_b. These methods may enable calculation of the reliability coefficient for Likert-ordered items. For example, the average Spearman inter-item correlation coefficient was 0.35, leading to an estimated alpha coefficient of 0.729. However, due to the multiple steps required to estimate ordinal reliability coefficients with SPSS, the use of alternative programs is strongly recommended.

The estimated ordinal alpha values obtained from various programs (omega, alpha with polychoric correlation matrix, structuralScale, and item.alpha) were consistent, each yielding a value of 0.76. This uniformity arises because all programs employ the same formula and polychoric correlation coefficient. The manually calculated value, using the estimated polychoric correlation coefficients, corroborated this result.

The results of the nonlinear SEM reliability estimation [10,11] included both the ordinal alpha computed using the method of Zumbo et al. [2] and compReSEM, with the tau.eq=TRUE option applied with and without the ord.scale option. To facilitate comparison of the estimated ordinal alpha coefficient, the tau.eq option was set to TRUE. The estimated coefficient was 0.76—identical to the ordinal alpha coefficient—when scale variance was unadjusted (ord.scale=FALSE) and 0.7 with the ord.scale=TRUE option was applied. When tau.eq=FALSE, the estimated reliability coefficient represents the omega value.

The application of a nonparametric correlation estimation method, namely Spearman correlation, to the reliability alpha formula yielded an estimated coefficient of 0.73. However, when applying the Kendall tau_b correlation, the coefficient was 0.68.

Results estimated using the DCER method indicate that the reliability coefficient varies based on the method used to estimate item-score correlation. When the ratio of maximum correlation between an item and the total score was employed, the alpha reliability estimate was 0.72. Utilizing gamma correlation statistics, the estimated alpha reliability coefficient was 0.78, compared to 0.72 when using the Sommer D.

The estimated IRT reliability coefficient was 0.74, as determined using the empirical_rxx function from the mirt program and the irtreliability function from the irtreliability program.

The estimated values for Likert scale reliability using the KR-20 and KR-21 methods [17] were 0.7039 and 0.7016, respectively. The results are consistent with those obtained using the reliability alpha coefficient, differing from ordinal alpha and other alternative methods. However, a limitation of this approach is the current unavailability of software for the program.

## Conclusion

Despite reported limitations of the reliability alpha and alternative reliability estimation methods, approaches to estimating reliability for the ordinal Likert scale remain limited. Since the introduction of the ordinal alpha method in 2009, its limitations, as well as those of alternative methods like the nonlinear SEM reliability estimation method, have been acknowledged. However, other approaches, such as the nonparametric DCER and the IRT-based reliability estimation method, have not been widely applied to Likert scales.

Until all available estimation methods are fully validated and accessible to nursing researchers, it is recommended to report not only the traditional Cronbach’s alpha but also the ordinal alpha. The consequences of biased reliability estimation extend beyond the development of instruments, as the scales from these instruments may also influence the accuracy of statistical evaluations, including even simple bivariate correlations or two-group comparisons. While adopting a single universally accepted reliability coefficient (such as Cronbach’s alpha) may be the preferred practice for a given type of scale, the publication of multiple articles discussing the limitations of Cronbach’s alpha and alternative reliability coefficients has prompted increased recognition of the complexities involved in measuring reliability.

Likert-scale instruments are commonly employed in nursing research, making their evaluation essential. While reports have described limitations of and alternatives to the application of the reliability alpha, these have not substantially clarified how the distinct attributes of the ordinal categorical scale may influence the reliability evaluation practices of nursing researchers.

The introduction of ordinal alpha by Zumbo et al. [2] has highlighted additional possibilities, prompting nurse researchers to anticipate the development of a new universally accepted reliability indicator. Several studies have recommended decision-making frameworks for identifying suitable reliability estimation methods for Likert scales [6,8,9]. These guidelines could assist nurse researchers in selecting the appropriate reliability estimation method and in reporting their findings, thereby enhancing the comprehension of the instruments used.

Best practices involve reporting a variety of reliability coefficients to gain a comprehensive understanding of the instrument used. This broader perspective can assist nursing researchers in making more informed decisions to improve the instrument. Relying on a single reliability coefficient can lead to misguided development; therefore, a more informed approach to decision-making is necessary and should be applied in further research.

## Figures and Tables

**Table 1.** Demonstration of ordinal reliability estimation methods with sample data

Category	Function	Description	Alpha
Package			
jamovi syntax	ordinal reliability		0.76
psych	omega	>omega(data, poly=TRUE)	0.76
	alpha	>poly<-polychoric(data)	0.76
		>alpha(poly$rho)	
ufs	structuralScale	>structualScale(data,ordered=TRUE)	0.76
misty	item.alpha	>item.alpha(data, ordered=TRUE)	0.76
SEM methods			
lavaan	reliability	>fit<-cfa(“F=~A1+A2+A3+A4+A5”,data, ordered=T)	
semTools	compReISEM		
		>reliability(fit)	0.76
		>compReSEM(fit,tau.eq=T,ord.scale=F)	0.76
		>compReSEM(fit, tau.eq=T,ord.scale=T)	0.70
Nonparametric methods			
R psych & stats	cor	>cor<-cor(data,method=”spearman”)	0.73
		>alpha(cor)	
	alpha	>cor<-cor(data,method=”Kendall”)	0.68
		>alpha(cor)	
DCER^†^		Rit (max. item-score correlation ratio)	0.72
		gamma	0.78
		Sommer D	0.72
IRT methods			
mirt	mirt	>re<-mirt(data,1,"gpcm", SE = TRUE)	
	fscores	>theta<-fscores(re,fullscores.SE=TRUE)	0.74
	empirical_rxx	>empirical_rxx(theta)	
irtreliability	irtreliability	> irtreliability(re, "GPCM", rep(6, 5), relcoef = "mrc")	0.74

DCER, deflation-corrected estimators of reliability; IRT, iItem response theory; PPDA, psychometrics & post-data analysis; SEM, structural equation modeling.

† Only an Excel worksheet demonstration is available. The estimated alpha is provided for comparison purposes.
